# Efficient Calculation Methods for the Diffusion Coefficient of Interstitial Solutes in Dilute Alloys

**DOI:** 10.3390/ma12091491

**Published:** 2019-05-08

**Authors:** Xiaoshuang Wang, Jürgen Faßbender, Matthias Posselt

**Affiliations:** 1 Institute of Ion Beam Physics and Materials Research, Helmholtz-Zentrum Dresden–Rossendorf, 01328 Dresden, Germany; j.fassbender@hzdr.de (J.F.); m.posselt@hzdr.de (M.P.); 2 Institut für Festkörper- und Materialphysik, Technische Universität Dresden, 01062 Dresden, Germany

**Keywords:** diffusion coefficient, interstitial solute, dilute alloy, efficient calculation, first-principle calculations, atomistic kinetic Monte Carlo simulations

## Abstract

In the example of oxygen diffusion in dilute ferritic iron alloys it is shown that the calculation of the diffusion coefficient can be separated into a contribution related to the migration in the interaction region between oxygen and the substitutional solute and a part related to diffusion in pure body centered cubic (bcc) Fe. The corresponding diffusion times are determined by analytical expressions using Density-Functional-Theory (DFT) data for the respective binding energies. The diffusion coefficient in the interaction region must be determined by atomistic kinetic Monte Carlo (AKMC) simulations with DFT values for the migration barriers as input data. In contrast to previous calculations, AKMC simulation must only be performed for one concentration of the substitutional solute, and the obtained results can be employed to obtain data for other concentrations in a very efficient manner. This leads to a tremendous decrease of computational efforts. Under certain conditions it is even possible to use analytical expressions where merely DFT data for the binding energies are needed. The limits of applicability of the presented calculation procedures are discussed in detail. The methods presented in this work can be generalized to interstitial diffusion in other host materials with small concentrations of substitutional solutes.

## 1. Introduction

Solutes with an atomic size smaller than that of the host atoms migrate via the interstitial mechanism [1]. In this case the most stable site of the solute and the saddle point for the migration are highly symmetric interstitial positions in the lattice, e.g., octahedral and tetrahedral sites. Foreign atoms with atomic sizes similar to or larger than those of the solvent occupy substitutional sites. The presence of native defects such as vacancies and self-interstitial atoms is generally a prerequisite for their migration. The diffusion via the vacancy and the interstitialcy (or indirect interstitial) mechanism is much slower than that of the interstitial solutes, since the concentration of the native point defects is rather low under the conditions of thermal equilibrium. Therefore, in calculations of the diffusion coefficient of interstitial solutes in dilute alloys, pre-existing substitutional solutes can be assumed to be immobile. By definition, in a dilute alloy the migration of the diffusing interstitial atom cannot be influenced at the same time by more than one substitutional solute.

In this work, efficient methods for calculating the diffusion coefficient of an interstitial solute in dilute ferritic iron alloys under the influence of substitutional foreign atoms are presented. Typical interstitial diffuser in bcc Fe are C, N, O, He, and H. Present investigations are focused on oxygen diffusion. In pure bcc Fe the most stable site of oxygen is the octahedral site and the most probable migration path is a first-neighbor jump between two octahedral sites, with the saddle point at a tetrahedral position [2]. Recently, the effect of various substitutional solutes on oxygen diffusion in bcc Fe was investigated using a combination of Density Functional Theory (DFT) calculations and atomistic kinetic Monte Carlo (AKMC) simulations on a rigid lattice [2]. DFT was applied to determine both the binding energy between oxygen and different substitutional atoms and the respective migration barriers in the vicinity of those solutes. Although the migration barriers are often rather different to that in pure bcc Fe, in most cases the relevant migration paths are first-neighbor jumps between modified (nearest neighbor) octahedral sites with modified tetrahedral sites as saddle points. The oxygen diffusion coefficient, as function of temperature and concentration of the substitutional solute, was obtained from AKMC simulations with the migration barriers from DFT calculations as input data. It was found that the deviation of the diffusion coefficient of oxygen in the dilute alloy from that in pure bcc Fe becomes more pronounced with increasing attraction between oxygen and the substitutional atom. Such deviations can be significant, even at concentrations of the substitutional solute below 0.1%.

## 2. Calculation Methods

In the present work the DFT data for binding energies and migration barriers determined in Reference [2] are used. In that paper spin-polarized DFT calculations were performed using the Vienna ab initio simulation package (VASP) [3,4,5] with a plane-wave cutoff energy of 500 eV. Electron-core interaction was described by projector-augmented wave (PAW) pseudopotentials [6,7] and exchange and correlation effects were treated within the framework of the generalized gradient approximation (GGA) using the Perdew-Burke-Ernzerhof (PBE) functional [8]. A supercell with 128 bcc lattice sites was considered, and Brillouin-zone sampling was performed using a 3 × 3 × 3 k point grid within the Monkhorst-Pack scheme [9]. The integration in reciprocal space was done with a Methfessel-Paxton smearing width of 0.2 eV [10].

**Figure 1:**
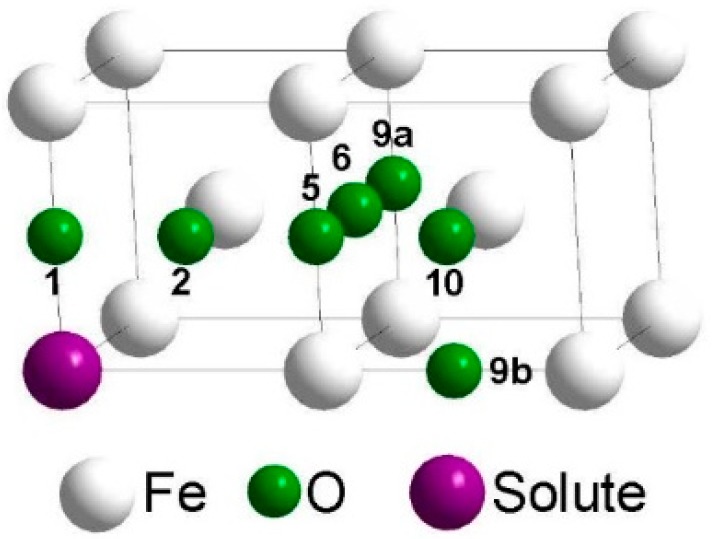
Denotation of octahedral interstitial sites in the neighborhood of a substitutional solute (up to 10th neighbor shell).

**Figure 2:**
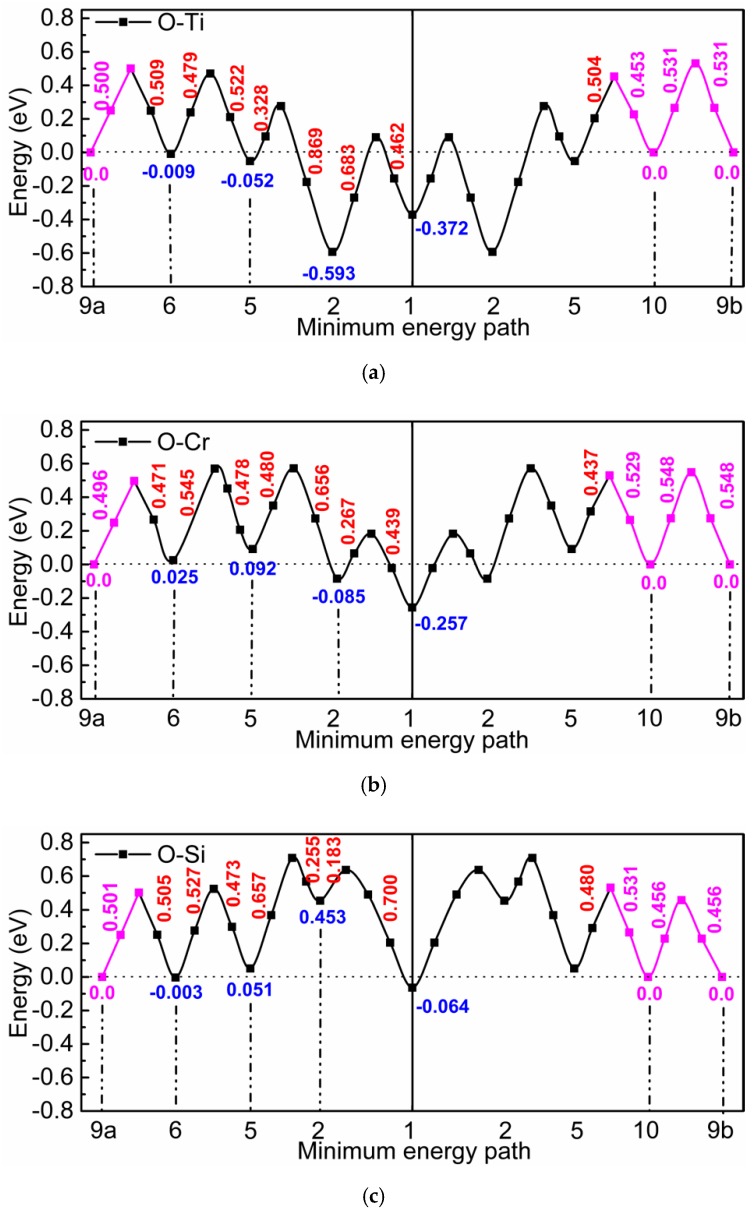
Migration barriers for oxygen in the environment of Ti (**a**), Cr (**b**), and Si (**c**). The red and blue numbers show the barrier height and the binding energy, respectively. The magenta lines and numbers show modifications of the original Density-Functional-Theory (DFT) data according to the rule of detailed balance (see text).

Figure 1 shows how many oxygen octahedral interstitial sites were considered in the environment of a substitutional foreign atom. The notation of the neighbor positions is according to the scheme for a simple cubic lattice (see [11]), which consists of the bcc lattice sites and the octahedral interstitial sites of the bcc lattice. Within the framework of this scheme oxygen cannot reside on third, fourth, seventh, eighth, etc. neighbor positions of the substitutional solute, since these sites are already occupied by iron atoms, and there are two different ninth neighbor sites (9a and 9b). After introduction of an oxygen and a foreign atom on the respective sites the positions of all atoms and the volume and shape of the supercell were relaxed, until the residual force on each atom became lower than 10^−2^ eV/Å and the change of total energy during self-consistent energy minimization for given atomic positions fell below 10^−5^ eV. Figure 2 illustrates three characteristic results of Reference [2]: (i) In the environment of Ti, strong attractive states exist for oxygen at the 1st and 2nd neighbor distance, and in this region the barriers are relatively high compared to those in perfect bcc Fe (0.512 eV). (ii) In the interaction region with Cr the attraction is weaker and the barriers are somewhat lower. (iii) Very weak attraction and repulsion dominates in the region near a Si atom and the migration barriers are rather different. In Figure 2 the interaction between oxygen and the substitutional solute extends up to the 10th neighbor shell. For use in AKMC simulations the original DFT data for barriers were modified according to the rule of detailed balance since the binding energy at neighbors 9a, 10, and at sites outside the 10th neighbor shell must be zero. Furthermore, the binding energy at neighbor 9b was also set to zero and the detailed balance was applied to change the respective barrier. Details of the AKMC simulations which are based on the residence time algorithm were described in Reference [2].

In all cases considered in that paper the AKMC simulation cell contained one diffusing oxygen atom and one substitutional solute. In order to model the concentration of substitutional foreign atoms, in Reference [2] separate AKMC simulations were performed for different cell sizes. In the following it is shown that in general AKMC simulations are only required for one concentration (or cell size), and the obtained results can be employed to obtain data for other concentrations in a very efficient manner.

At a given temperature and concentration of substitutional foreign atoms the diffusion coefficient of oxygen (or of another interstitial solute) can be written as

(1a) $$D=\frac{t_{\mathrm{free}}}{t_{\mathrm{total}}}D_{\mathrm{free}}+\frac{t_{\mathrm{inter}}}{t_{\mathrm{total}}}D_{\mathrm{inter}}$$

with

(1b) $$t_{\mathrm{total}}=t_{\mathrm{free}}+t_{\mathrm{inter}}$$

where $D_{\mathrm{free}}$ is the diffusion coefficient of oxygen in pure bcc Fe, i.e., outside the region of influence by the substitutional solute, and $D_{\mathrm{inter}}$ is the diffusion coefficient of oxygen inside the interaction region. The quantities $t_{\mathrm{free}}$ and $t_{\mathrm{inter}}$ correspond to the sum of the time periods for diffusion outside or inside the region of influence, respectively, and $t_{\mathrm{total}}$ is the total diffusion time. The value of $D_{\mathrm{free}}$ is given by the known analytical formula

(2) $$D_{\mathrm{free}}=\frac{a^{2}}{6}\nu_{0}^{free}\exp\left(\frac{-E_{m}^{free}}{k_{B}T}\right)$$

with the lattice constant a, as well as the migration barrier $E_{m}^{free}$ and the attempt frequency $\nu_{0}^{free}$ in pure bcc Fe, with a=2.832Å, $E_{m}^{free}=0.512\;\;\mathrm{eV}$, and $\nu_{0}^{free}=15.76\;\;\mathrm{THz}$ [2]. The values of $t_{\mathrm{free}}$, $t_{\mathrm{inter}}$, and $t_{\mathrm{total}}$ may be obtained from AKMC simulations as performed in Reference [2] for different solute concentrations or cell sizes. However, the time ratios in Equation (1a) can be also expressed by analytical relations containing terms with probabilities for a certain interaction of oxygen with the substitutional solute.

(3a) $$\frac{t_{\mathrm{inter}}}{t_{\mathrm{total}}}=\frac{\sum_{i}N_{i}\exp\left(-\frac{E_{bind}^{i}}{k_{B}T}\right)c_{S}}{1-\sum_{i}N_{i}c_{S}+\sum_{i}N_{i}\exp\left(-\frac{E_{bind}^{i}}{k_{B}T}\right)c_{S}},\;i=1,2,5,6,9a,9b,10$$

(3b) $$\frac{t_{\mathrm{free}}}{t_{\mathrm{total}}}=\frac{1-\sum_{i}N_{i}c_{S}}{1-\sum_{i}N_{i}c_{S}+\sum_{i}N_{i}\exp\left(-\frac{E_{bind}^{i}}{k_{B}T}\right)c_{S}}$$

$\left(N_{1}=2,\;N_{2}=4,\;N_{5}=8,\;N_{6}=8,\;N_{9a}=8,\;N_{9b}=2,\;N_{10}=8\right)$

These relations are based on the Gibbs distribution of the probability to find the system with the oxygen atom and the substitutional solute in a particular state (see Reference [12]). The quantity $E_{bind}^{i}$ denotes the binding energy of the pair at the ith neighbor distance, see Figure 1. The quantity $c_{S}$ is the concentration of the substitutional solute, and $N_{i}$ is the possible number of substitutional solute sites in the ith neighborhood of oxygen. In calculations of the time ratios it is taken into account that at neighbors 9a, 9b, 10, and at sites outside the 10th neighbor shell (see Figure 1) the binding energy has to be zero. It can be assumed that $D_{\mathrm{inter}}$ is nearly independent of the concentration of the substitutional solute (or the size of the AKMC simulation cell) since this quantity is only determined by migration paths inside the region of influence by the substitutional solute. In this case AKMC simulation needs to be used only once, i.e., for a certain concentration of the substitutional foreign atom, and the total diffusion coefficient D can be then determined for the other concentrations using Equations (1)–(3). It is quite clear that such a method is much more efficient than performing separate AKMC simulations for different concentrations or cell sizes as done in Reference [2].

## 3. Results and Discussion

### 3.1. The Value of $D_{\mathrm{inter}}$

**Figure 3:**
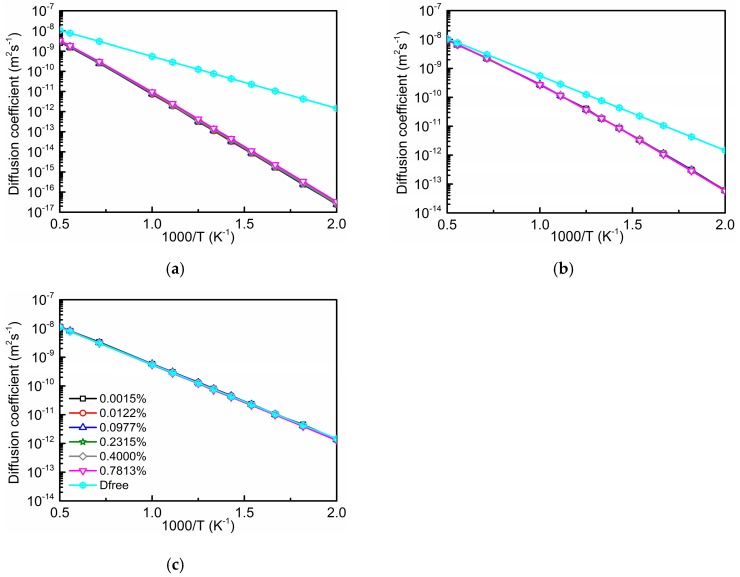
Diffusion coefficients of oxygen inside ($D_{\mathrm{inter}}$) and outside ($D_{\mathrm{free}}$) the interaction region with Ti (**a**), Cr (**b**), and Si (**c**), for different concentrations $c_{S}$ (in at.%) of these substitutional solutes.

Figure 3 depicts data of $D_{\mathrm{inter}}$ determined by AKMC simulations for different cell sizes (concentrations of the substitutional foreign atoms): 32 × 32 × 32 bcc unit cells (0.0015%), 16 × 16 × 16 unit cells (0.0122%), 8 × 8 × 8 unit cells (0.0977%), 6 × 6 × 6 unit cells (0.2315%), 5 × 5 × 5 unit cells (0.4000%), and 4 × 4 × 4 unit cells (0.7813%), together with the oxygen diffusion coefficient $D_{\mathrm{free}}$ in pure bcc Fe. Ti, Cr, and Si substitutional solutes were considered and their influence on the diffusing oxygen is taken into account up to the 10th neighbor shell, see Figure 1.

The value of $D_{\mathrm{inter}}$ is indeed nearly independent of concentration. The more attractive the interaction of oxygen with a substitutional solute is, the larger is the difference between $D_{\mathrm{inter}}$ and $D_{\mathrm{free}}$, i.e., the lower is the value of $D_{\mathrm{inter}}$. The Arrhenius plots show almost straight lines for $D_{\mathrm{inter}}$ since this quantity is related to migration paths where the diffusing oxygen atom passes repeatedly the interaction region. However, the motion of oxygen on these paths does not correspond to a migration in a lattice with an exactly periodic sequence of barriers. Therefore, slight deviations from a straight line are found (see Figure 3b). At this point it should be noticed that the data presented in this work are only valid for ferromagnetic iron, i.e., below the Curie temperature (1043 K). One must also note that at about 1183 K the α (bcc) to γ (fcc) transition occurs. The figures show a temperature scale up to 2000 K in order to verify that the total diffusion coefficient of oxygen approaches the value for pure bcc Fe at sufficiently high temperatures (see Figure 6). It is also worth mentioning that the temperature dependence of the spontaneous magnetization in ferromagnetic iron is not considered in the present study, i.e., for bulk iron always the ground state value of magnetization is assumed.

**Figure 4:**
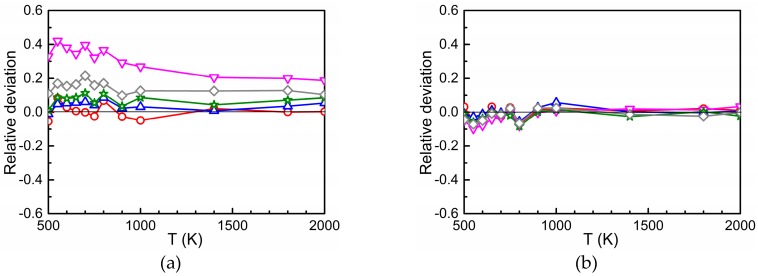

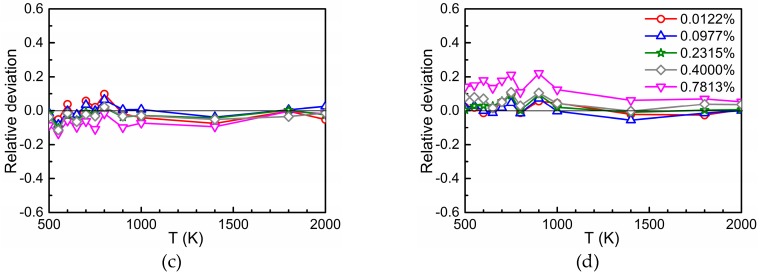
Relative deviation $\left[D_{\mathrm{inter}}(c_{S})-D_{\mathrm{inter}}(c_{S}^{0})]/D_{\mathrm{inter}}(c_{S}^{0}\right)$ for different values of $c_{S}$ in the case of Ti (**a**), Cr (**b**), and Si (**c**) in dependence on temperature ($c_{S}^{0}$ = 0.0015%). If the interaction region between oxygen and Ti is reduced to the 5th neighbor shell the deviations become smaller (**d**).

Figure 4a–c show the relative deviation of $D_{\mathrm{inter}}$ at concentrations $c_{S}$ of 0.0122%, 0.0977%, 0.2315%, 0.4000%, and 0.7813% from that at $c_{S}^{0}=0.0015\%$, i.e., the quantity $\left[D_{\mathrm{inter}}(c_{S})-D_{\mathrm{inter}}(c_{S}^{0})]/D_{\mathrm{inter}}(c_{S}^{0}\right)$. In general, the relative deviations are small which is in accord with Figure 3 where a logarithmic scale was used on the ordinate. However, the difference found for the two highest concentrations (0.7813 and 0.4000%) of the substitutional solute Ti cannot be explained by statistical fluctuations of the AKMC results. Obviously, in these cases the size of the AKMC simulation cell is too small to achieve a sufficient “randomization” of the trajectories of the migrating oxygen between successive passes through interaction regions. In other words, the entrance and exit points for migration to and from different interaction regions (or for migration in pure Fe) are slightly correlated, which does not occur at low concentration. In such a case the entrance points to a subsequent interaction region may be not fully randomly distributed over the interface between this region and pure Fe. This issue was considered in more detail by performing AKMC simulations for the case that the interaction region of oxygen with the substitutional foreign atom is reduced to the 5th neighbor shell. For this purpose, outside the 5th neighbor shell the binding energies were set to zero and the corresponding barriers were set equal to the migration energy in pure bcc Fe, furthermore the rule of detailed balance was applied in the transition region. Figure 4d clearly shows that for 0.4000 and 0.7813% Ti the relative deviation is smaller than in Figure 4a. Obviously, in the case of Figure 4d more space is available for the “randomization” of the trajectories in pure Fe. The results presented in Figure 4 show that in cases of strong attraction between O and the substitutional solute a minor dependence of $D_{\mathrm{inter}}$ on concentration occurs at higher concentration values, whereas this is not observed for weak attraction or repulsion. This minor dependence is stronger at low temperature.

### 3.2. Time Ratios

**Figure 5:**
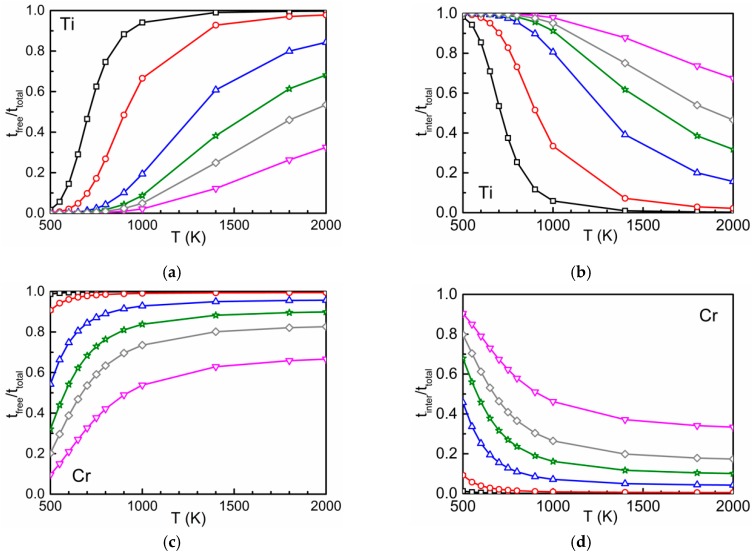

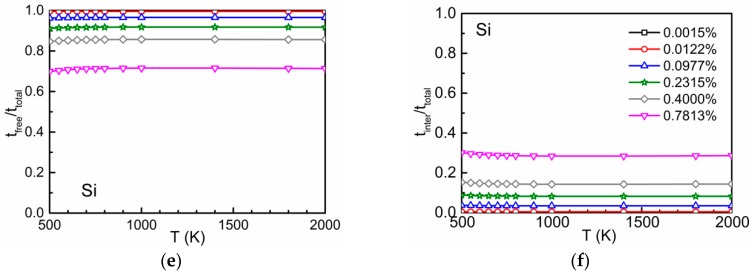
The time ratios $t_{\mathrm{free}}/t_{\mathrm{total}}$ (left) and $t_{\mathrm{inter}}/t_{\mathrm{total}}$ (right) in dependence on temperature and concentration for different concentrations of Ti (**a**,**b**), Cr (**c**,**d**) and Si (**e**,**f**).

The time ratios $t_{\mathrm{free}}/t_{\mathrm{total}}$ and $t_{\mathrm{inter}}/t_{\mathrm{total}}$ according to Equation (3) are shown in Figure 5. Due to the attraction between oxygen and Ti and between oxygen and Cr at the first- and second-neighbor distances, at low temperatures the residence time of the diffusing atom in the interaction region is relatively high [2]. This leads to high values for $t_{\mathrm{inter}}/t_{\mathrm{total}}$ and low values for $t_{\mathrm{free}}/t_{\mathrm{total}}$. The opposite behavior is found at high temperatures. The higher the concentration of the substitutional solute the lower (higher) are the values of $t_{\mathrm{free}}/t_{\mathrm{total}}$ ($t_{\mathrm{inter}}/t_{\mathrm{total}}$) at a given temperature. On the other hand, the interaction between oxygen and Si is repulsive and weakly attractive. Therefore, $t_{\mathrm{inter}}/t_{\mathrm{total}}$ ($t_{\mathrm{free}}/t_{\mathrm{total}}$) is low (high) within the whole temperature range.

A verification that the ratios $t_{\mathrm{free}}/t_{\mathrm{total}}$ and $t_{\mathrm{inter}}/t_{\mathrm{total}}$ determined by Equation (3) are equivalent to those calculated by separate AKMC simulations for different concentrations of the substitutional solutes is also important. It was found that the relative deviation of the AKMC data from those determined by Equation (3) is below about 2% in the temperature range between 500 and 2000 K, for all concentrations of Ti, Cr, and Si considered. These results justify the use of Equation (3), in combination with Equations (1) and (2), to determine the total oxygen diffusion coefficient at given concentration and temperature.

### 3.3. Total Diffusion Coefficient

**Figure 6:**
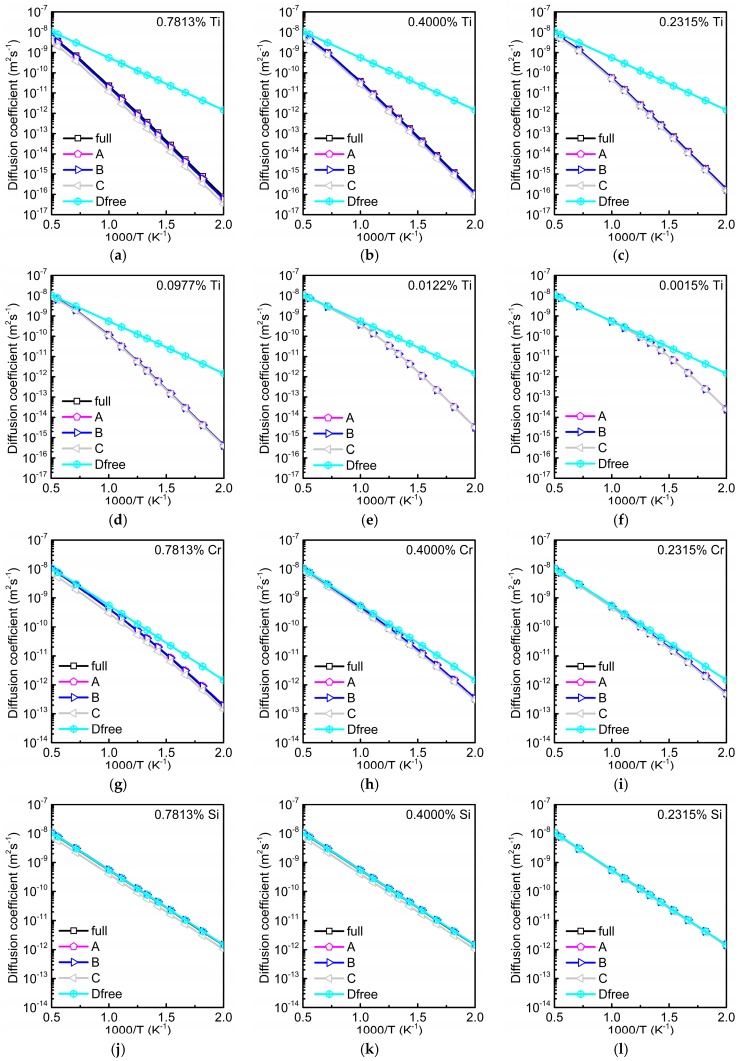
Total diffusion coefficient D of oxygen in dependence on temperature and solute concentration obtained by different calculation methods. Curves marked by ‘full’ show results from full atomistic kinetic Monte Carlo (AKMC) simulations [2]. ‘A’ denotes the data determined by Equations (1) and (2) with the time ratios and $D_{\mathrm{inter}}$ from AKMC simulations for the respective concentrations $c_{S}$. Data determined by Equations (1)–(3) with $D_{\mathrm{inter}}$ from AKMC simulations are marked by ‘B’, while results from Equation (4) with $t_{\mathrm{free}}/t_{\mathrm{total}}$ from Equation (3) are denoted by ‘C’. The diffusion coefficient of oxygen in pure bcc Fe ($D_{\mathrm{free}}$) is plotted as reference. For Cr or Si below 0.2315% the difference between D and $D_{\mathrm{free}}$ is very small. Therefore, these data are not shown. Results for Ti (**a**–**f**), for Cr (**g**–**i**), and for Si (**j**–**l**).

Data for the total diffusion coefficient D of oxygen in different dilute alloys are depicted in Figure 6. They were calculated using four different methods: (i) by full AKMC simulations as in Reference [2], (ii) by Equations (1) and (2), i.e., with $D_{\mathrm{inter}}$, $t_{\mathrm{free}}/t_{\mathrm{total}}$ and $t_{\mathrm{inter}}/t_{\mathrm{total}}$ from AKMC simulations, (iii) by Equations (1)–(3), i.e., with a concentration-independent value of $D_{\mathrm{inter}}$ from AKMC simulations (taken from Figure 3 for the concentration of 0.0977%), and (iv) using the equation

(4) $$D=\frac{t_{\mathrm{free}}}{t_{\mathrm{total}}}D_{\mathrm{free}}$$

with $t_{\mathrm{free}}/t_{\mathrm{total}}$ from Equation (3a).

In Equation (4) merely DFT binding energy data and the migration barrier of oxygen in pure bcc Fe are required. Figure 6 clearly shows that the data obtained by methods (i–iii) are more or less equal for the concentrations of substitutional solutes considered. It must be also stressed that the slight concentration dependence of $D_{\mathrm{inter}}$ found at higher fractions of Ti is not visible in the Arrhenius plot for D (Figure 6). In many cases, especially for low concentrations, even the data obtained by all the four methods agree very well. Under this condition D can be easily obtained by Equation (4) for which the knowledge of the binding energies $E_{bind}^{i}$ and $D_{\mathrm{free}}$ is sufficient. This kind of calculation does not require any AKMC simulation. In the example of the substitutional solute Ti the data for D in the Arrhenius plot show deviations from a straight line for concentrations below about 0.2315%. In this case the second term of Equation (1a) is generally smaller than the first one so that temperature dependence is only determined by $t_{\mathrm{free}}/t_{\mathrm{total}}$ times $D_{\mathrm{free}}$, i.e., by Equation (4). If the interaction with oxygen is repulsive and weakly attractive as in the case of Si, $D_{\mathrm{inter}}$ is nearly equal to $D_{\mathrm{free}}$ (see Figure 3) and for higher concentrations both $t_{\mathrm{inter}}/t_{\mathrm{total}}$ and $t_{\mathrm{free}}/t_{\mathrm{total}}$ must be taken into account (see Figure 5). Therefore, in Figure 6 the results obtained by Equation (4) slightly differ from the others at concentrations of 0.7813 and 0.4000%. At lower concentrations D becomes more or less equal to $D_{\mathrm{free}}$.

Only few experimental data on the oxygen diffusion coefficient in bcc Fe are available. They were measured many years ago using the method of internal oxidation of solutes which have a higher affinity to oxygen than iron [13,14,15]. The experiments were performed between 1023 and 1173 K, i.e., mainly in paramagnetic state. Data for the diffusion coefficient of oxygen in the iron matrix were estimated by extrapolation to zero volume fraction of the formed oxide. The values obtained in such a manner were considerably lower than those of $D_{\mathrm{free}}$ and show no significant dependence on the kind of solute. Such a slow diffusion may be due to strong traps which are not considered in this work. The vacancy could be such a trap since it was recently shown that the presence of oxygen increases the total vacancy concentration considerably [16]. Detailed investigations on these issues should be the subject of future work. Thereby new experimental investigations with up-to date analytical tools may be very helpful.

## 4. Conclusions

In conclusion, effective procedures to determine the diffusion coefficient of the interstitial solute oxygen in dilute iron alloys have been presented and the limits of their applicability are discussed. Compared to previous calculations on carbon [17,18] and oxygen [2] diffusion under the influence of foreign atoms on substitutional sites, AKMC simulation must be performed only for one concentration of the substitutional solute which leads to a considerably shorter computing time. For sufficiently low concentrations of solutes (below 0.2315 at.%), it is even possible to determine the diffusion coefficient of oxygen by an analytical expression where only the diffusion coefficient of oxygen in pure bcc Fe and the binding energy between oxygen and solutes at different neighbor distances are needed, i.e., no further AKMC calculation is required. The calculation methods described in this work can be used for other interstitial diffusers in dilute iron alloys and also for interstitial diffusion in other host materials with small concentrations of substitutional solutes.
